# MicroRNA screening identifies miR-134 as a regulator of poliovirus and enterovirus 71 infection

**DOI:** 10.1038/sdata.2017.23

**Published:** 2017-03-01

**Authors:** Nichole Lynn Orr-Burks, Byoung-Shik Shim, Weilin Wu, Abhijeet A. Bakre, Jon Karpilow, Ralph A. Tripp

**Affiliations:** 1 Department of Infectious Diseases, College of Veterinary Medicine, University of Georgia, Athens, Georgia 30602, USA; 2 Proventus Bio, 220 Riverbend Rd, Athens, Georgia 30602, USA

**Keywords:** RNAi, High-throughput screening, miRNAs, Virology

## Abstract

MicroRNAs (miRNAs) regulate virus replication through multiple mechanisms. Poliovirus causes a highly debilitating disease and though global efforts to eradicate polio have sharply decreased polio incidence, unfortunately three countries (Afghanistan, Nigeria and Pakistan) remain polio-endemic. We hypothesize that understanding the host factors involved in polio replication will identify novel prophylactic and therapeutic targets against polio and related viruses. In this data set, employing genome wide screens of miRNA mimics and inhibitors, we identified miRNAs which significantly suppressed polio replication. Specifically, miR-134 regulates poliovirus replication via modulation of ras-related nuclear protein (RAN), an important component of the nuclear transport system. MiR-134 also inhibited other Picornaviridae viruses including EV71, a growing concern and a high priority for vaccination in Asian countries like China. These findings demonstrate a novel mechanism for miRNA regulation of poliovirus and other Picornaviridae viruses in host cells, and thereby may provide a novel approach in combating infection and a potential approach for the development of anti-Picornaviridae strategies.

## Background and Summary

Poliovirus (PV), a non-enveloped human enterovirus virus of the Picornaviridae family is the etiological agent of poliomyelitis (polio). Poliovirus symptoms include fever, fatigue, headache, limb pain and permanent paralysis in some cases. Global vaccination campaigns utilizing live-attenuated oral polio vaccines have reduced PV in all but three countries (Afghanistan, Nigeria and Pakistan) which still remain PV-endemic. The global use of live-attenuated vaccines has an increased risk of reversion to wild type and shedding by immune-deficient individuals^1,2^. Though vaccination is effective, treatment of polio is symptomatic. Enterovirus 71 (EV71), a relative of PV, is a major public health burden in the Western Pacific region associated with severe neurologic symptoms of hand, foot and mouth disease (HFMD), a disease most often contracted by children<5 years old^3^. More than 7 million cases of HFMD were reported in mainland China from 2008–2012. EV71 vaccination programs are challenged by high cost and vaccine availability^4^. Understanding host gene networks that regulate PV and/or EV71 replication, and development of agents against these targets is a promising strategy to prevent and treat PV while eliminating the issues of reversion and shedding.

RNA interference (RNAi) is an evolutionarily conserved pathway of post transcriptional gene regulation involving small double-stranded non-coding RNAs, microRNAs (miRNAs). The ‘guide’ strand contains a 6 nt ‘seed’ region that binds complementary miRNA recognition elements (MRE) within the 3′ untranslated regions (3′-UTR) of transcripts in a RNA induced silencing complex (RISC) causing degradation of transcripts or blocking gene translation^5,6^. PV and EV71 can modulate multiple host pathways including RNAi for their own replication^7–13^. The role of miRNAs in these processes is incompletely understood. To understand the role of miRNAs in viral replication, we undertook a high-throughput screen utilizing miRNA inhibitors and mimics to determine which miRNAs alter the replication of poliovirus and the related EV71.

HEp-2C cells were reverse-transfected with non-targeting siRNA, miRNA negative controls, or gene specific mimics for 48 h. The gene specific mimics corresponded to 1,208 known miRNAs in the human genome. Cytotoxicity was evaluated via CytoTox-Glo assay (Promega). Mimics which were not cytotoxic were evaluated. Transfected cells were either mock infected (no virus) or infected with Sabin-2 strains of PV (MOI 0.01) or EV71 (MOI 0.01). EV71 was examined in miRNA-transfected cells, but was not used as part of the entire library screen.

**Figure 1:**
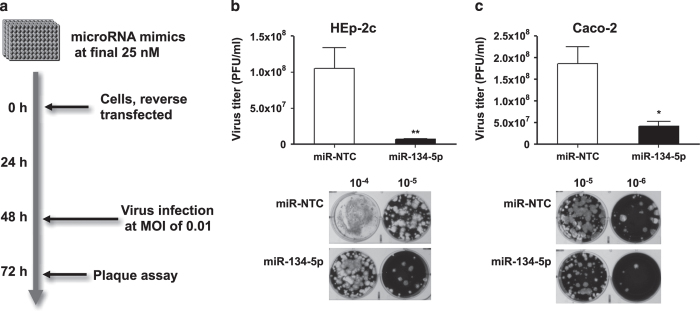
miR-134 inhibits sabin-2 replication in HEp-2c and Caco-2 cells. (**a**) Schematic of the experimental design. To evaluate if antiviral effect of miR-134 is cell type-specific, HEp-2c (**b**) and Caco-2 (**c**) cells were reverse-transfected with miR-134-5p mimic or miR mimic non-targeting scrambled negative control (miR-NTC) at a final concentration of 25 nM. After 48 h, the transfected cells were infected with Sabin-2 at MOI of 0.01 for 24 h and the virus titers were determined by plaque assay. Error bars represent s.e.m. and the significant differences are compared with miR-control. Each experiment was performed in quadruplicate and each experiment was repeated twice. The results were reproduced and the data in the figure were representative of 8 plates. *P<0.05, **P<0.01.

**Figure 2:**
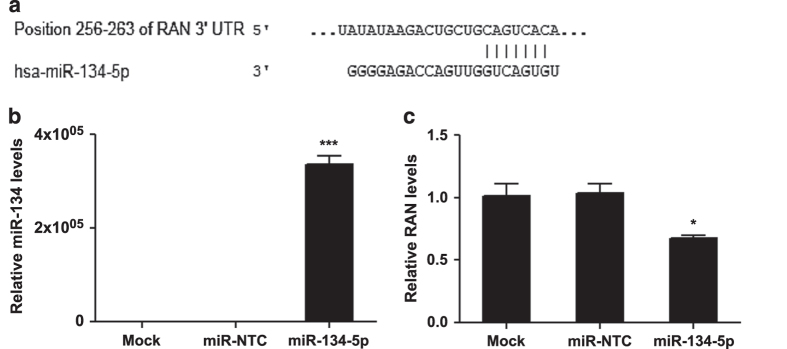
RAN is a target of miR-134. (**a**) Sequence of the putative binding site between miR-134 and 3′UTR of RAN. (**b**) and (**c**) Hep-2c cells were reverse-transfected with miR-134-5p or miR mimic non-targeting scrambled negative control (miR-NTC) at a final concentration of 25 nM. After 48 h, total RNA was isolated from HEp-2c cells, and then miR-134 (**b**) and RAN (**c**) mRNA levels were determined by qRT-PCR. The data were normalized to the 18S rRNA gene and were expressed as fold change relative to mock-transfected cells. The significant differences are compared with miR-control. Each experiment was performed in quadruplicate and each experiment was repeated twice. The results were reproduced and the data in the figure were representative of 8 plates. Error bars represent s.e.m. *P<0.05, ***P<0.001.

**Figure 3:**
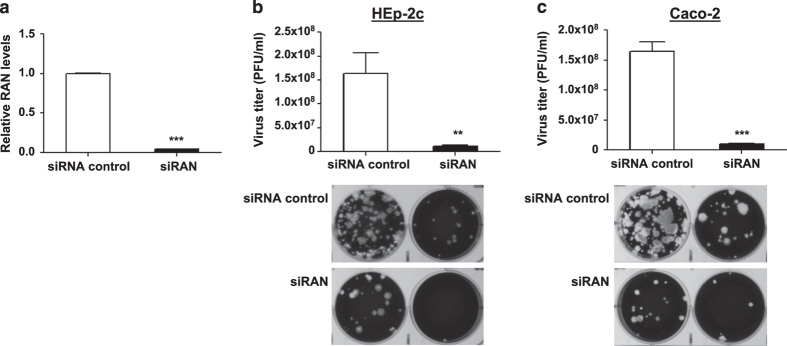
Silencing of RAN inhibits PV replication in HEp-2c and Caco-2 cells. The cells were reverse-transfected with siRAN or siRNA control at a final concentration of 25 nM. After 48 h, (**a**) total RNA was isolated from HEp-2c cells and RAN mRNA level was determined by qRT-PCR. The data were normalized to the 18S rRNA gene and were expressed as fold change relative to siRNA control. The transfected Hep-2c (**b**) and Caco-2 (**c**) cells were infected with Sabin-2 strain at an MOI of 0.01 for 24 h, and then the virus titers were determined by plaque assay. The significant differences are compared with siRNA control. Error bars represent s.e.m. of quadruplicate. Each experiment was performed in quadruplicate and each experiment was repeated twice. The results were reproduced and the data in the figure were representative of 8 plates. **P<0.01, ***P<0.001.

**Figure 4:**
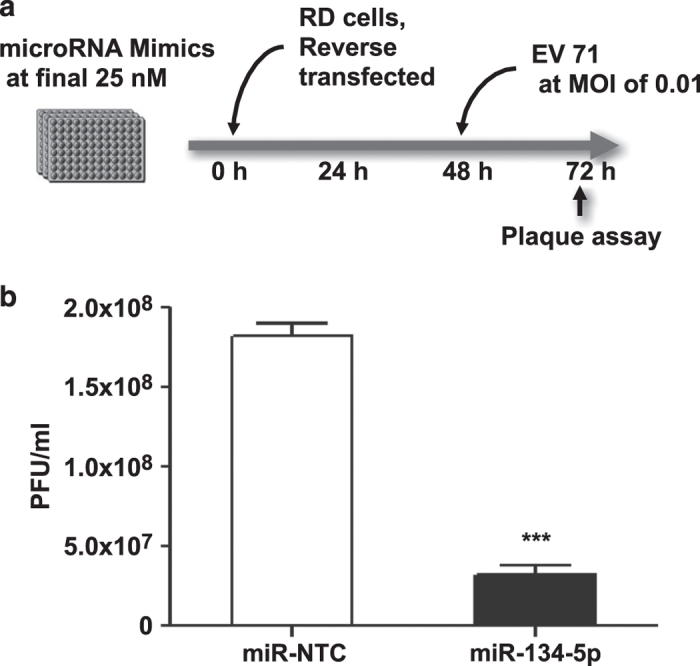
miR-134 targets EV71 replication in RD cells. (**a**) Schematic of the experimental design. (**b**) RD cells were reverse-transfected with miR-134-5p or miR mimic non-targeting scrambled negative control (miR-NTC) at final concentration of 25 nM. 48 h later the cells were infected with EV71 at MOI of 0.01 for 24 h and the antiviral effect of miR-134 was determined by plaque assay. Error bars represent s.e.m. of of quadruplicate. Each experiment was performed in quadruplicate and each experiment was repeated twice. The results were reproduced and the data in the figure were representative of 8 plates. The significant differences are compared with miR-control. ***P<0.001.

We investigated whether miRNA-transfection with miR-134 inhibited EV71 replication. Mimics/inhibitors against the *C. elegans* miR-67 were used as non-targeting controls while siRNAs targeting the PV capsid (siPol) were used as positive control. Twenty-four hours post-infection, cells were stored at −80 °C. miRNA effects on Sabin-2 replication were evaluated by a PV type-II specific ELISA^14,15^. Absorbance values were normalized to non-targeting miRNA mimic controls and converted to a z-score. Mimics that resulted in a z-score≤−1.5 s.d. were deemed hits and subjected to validation experiments which utilized miRNA inhibitors targeting the same genes (Table 1). Several miRNAs suppressed PV replication. MiR-134 and miR-138 had the most potent antiviral effect against PV Sabin-2 following transfection (Fig. 1). For this reason, the antiviral effects of miR-134 were evaluated further and the resulting data is outlined here. MiR-138 is currently still under evaluation. Ingenuity pathway analysis (IPA; QIAGEN) was utilized to evaluate protein networks associated with PV replication to predict targets of miR-134 that may be involved in PV replication. TargetScan analysis was used to predict target transcripts via seed region complementarity. IPA and TargetScan analysis revealed many predicted targets, of interest was the nuclear transport system protein (RAN) because it miR-197 inhibited EV72 replication^16^. We confirmed by qPCR of RAN mRNA and miR-134 transcript levels following miR-mimic transfection (Fig. 2). To confirm RAN’s role in PV replication, RAN was silenced via siRNA i prior to infection with Sabin-2 PV. Silencing inhibited PV replication in both cell lines (Fig. 3). miR-134 also drastically reduced EV71 replication (Fig. 4). It is also possible that miR-134 may regulate other host genes required for PV replication, which is supported by the evidence that one miRNA can regulate large numbers of target mRNAs^17^. Hits from primary screen were validated using miRNA specific inhibitors. These findings demonstrate miRNA regulation of PV and EV71 and may provide a novel approach in combating infection.

## Methods

### Cell culture and viruses

HEp-2 is an adherent human epidermoid carcinoma cell line that support PV replication. Caco-2 cells, a colorectal adenocarcinoma (HTB-37) adherent epithelial cell line derived from human colon tissue, were used in conjunction with HEp-2 cells to evaluate the antiviral effects of miR-134 against PV replication. Effects of target gene silencing on poliovirus replication can be detected 24–48 h post-infection (pi), and were utilized for the miRNA screen. Human muscle rhabdomyosarcoma (RD) cells are an adherent cell which supports enterovirus 71 (EV 71) replication and are routinely utilized to determine enterovirus titers post-miRNA transfection. All cell lines were cultured under the same conditions. All experiments were completed with cells at the same passage. As part of this study we investigated whether miRNA-transfection with miR-134 also inhibited replication of EV71, a familial relative of PV, in an effort to evaluate whether viruses within the same family exhibiting similar replication pathways would also be affected by miR-134. These experiments utilized the same negative control miRNA and siRNA as the PV evaluation^18^.

Briefly, cells were cultured in Dulbecco’s modified eagle medium (DMEM; Hyclone) supplemented with 10% heat-inactivated fetal bovine serum (FBS) under cell culture conditions (37 °C, 5% CO_2_). Cells were passaged 24 h prior to transfection. Briefly, cell culture media was removed and cells washed with 1X PBS to remove excess media and FBS. Following washing, cells were detached from the culture flask via addition and incubation with 0.05% Trypsin. Cells were incubated with trypsin under cell culture conditions until cells detached from the flask surface. Trypsin was neutralized with equal volumes of cell culture media containing 10% FBS. Trypsonized cells were removed from the cell culture flask and transferred to a 50 ml conical tube. Cell suspension was pelleted via centrifugation (1 min at 1,200×g). Supernatant was discarded and cells were resuspended in cell culture media containing 10% FBS by pipetting. Resuspended cells were then transferred back to the cell culture flask at the appropriate concentration and incubated under cell culture conditions. Master cell stocks were cultivated and stored at −80 °C. Passage numbers were kept consistent throughout all experiments to reduce variability. HEp-2Cand Caco-2 cells were utilized at P164 and P46, respectively.

The Sabin-2 PV vaccine strain was used throughout this study. This strain of PV is propagated in the African Green Monkey Kidney cell line Vero-P, the approved cell line for polio vaccine production. Cell stocks were obtained from the Centers of Disease Control and Prevention (CDC, Atlanta) who in turn obtained stocks from the Serum Institute of India (SI). Vero-P stocks were cultured under cell culture conditions at 37 °C, 5% CO_2_ in Dulbecco’s modified Eagle’s medium (DMEM; Hyclone, GE Healthcare) supplemented with 10% heat inactivated fetal bovine serum (FBS; Hyclone, GE Healthcare). Master cell stocks were cultivated and stored at −80 °C. Passage number were kept consistent throughout all experiments to reduce variability. Viral stocks were cultivated, viral titers determined by plaque assay and 50% cell culture infectious dose (CCID_50_), aliquoted and stored at −80 °C until use^19^.

### Primary screening and miRNA mimic transfection

The primary miRNA mimic screen included a library of 1,208 miRNA mimics (GE Dharmacon-Thermo Scientific; CS-001010 Human Mimics Lot 10100 and CS-001015 Supplement Human Mimic 16.0 Lot 11144). Screening was completed in triplicate using a 96-well plate format with HEp-2C cells (10^3^ cells per well). HEp-2C cells were reverse transfected to a final mimic concentration of 25 nM. The Dharmacon miRIDIAN microRNA mimic negative control (miR-NTC; CN-001000-01) was used as a negative control and a siRNA targeting the poliovirus capsid-coding region (siPol) (CDC, provided by Dr Steve Oberste) was utilized to monitor silencing effects on poliovirus replication in all assays. A cytoxicity transfection control siRNA (siTOX) (GE Healthcare Dharmacon TOX Transfection Control, Cat. # D0015000120) was utilized to visually confirm effective productive transfection. Cells successfully transfected with siTOX undergo apoptosis resulting in cell death. Briefly, mimics were incubated at room temperature for 20 min in 0.3% (0.3 ul per well) of DharmaFECT transfection reagent 4 (DF4) (Dharmacon-Thermo Scientific) prepared in serum-free medium (Opti-MEM, Invitrogen Inc.) prior to addition of cells in Dulbecco’s modified Eagle’s medium (DMEM, Invitrogen) supplemented with 10% calf serum. Cells were cultured for 48 h at 37 °C, 5% CO_2_. Forty-eight hours post-transfection, media was removed, cells were washed with phosphate buffer saline (PBS; Hyclone, GE Healthcare) and infected with Sabin 2 at an MOI=0.01 Virus was diluted in DMEM containing 2% calf serum and 1% penicillin-streptomycin. Twenty-four hours post-infection, HEp-2C cells were removed from the culture incubator and stored at −80 °C until evaluated.

### Validation screening and removal of false positives

Following the primary miRNA screen, in an effort to reduce false positives relative to transfection variability, individual miRNA inhibitors (GE Dharmacon Thermo-Fisher; IH-001010 Human Inhibitor Lot 09168 and IH-001015 Supplement Human Hairpin Inhibitor Library 16.0) were utilized to validate miRNA hits produced as part of the primary miRNA mimic screen. Hits producing an opposite phenotype as compared to the miRNA mimics screen were taken into future studies. The miRNA mimics are the focus of this study; therefore, the brief screening of the miRNA inhibitors was not included here and only used as a form of phenotype validation. Briefly, individual miRNA inhibitors corresponding to miRNA hits produced as part of the primary mimic screen were reverse-transfected into HEp-2 cells as previously described. At 48 h post-transfection, cells were infected with Sabin-1, Sabin-2 or Sabin-3 strains (MOI=0.01). Cells were incubated and replication assessed as previously stated. Messenger RNA levels for each gene were evaluated following transfection by qPCR.

**Figure 5:**
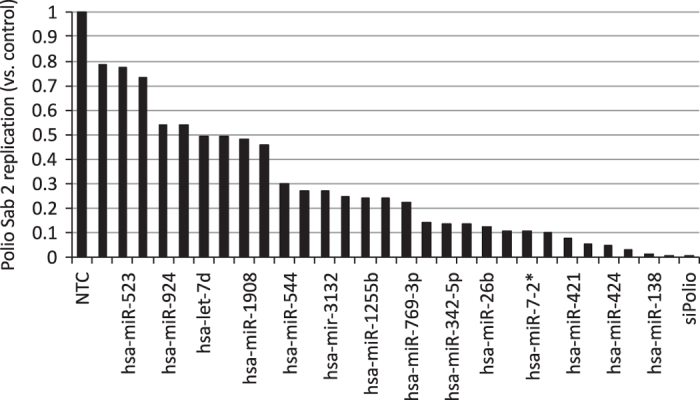
miRNAs inhibit sabin-2 replication in HEp-2c cells. Cells were reverse-transfected with miRNA mimic or miR-control mimic (miR-control) at a final concentration of 25 nM. After 48 h, the transfected cells were infected with Sabin-2 at MOI of 0.01 for 24 h and the virus titers were determined by ESLIA assay.

Validation screens were completed to further confirm the anti-virus effects of the identified miRNA mimics using ELISA assay. For the list of 108 anti-polio miRNA mimics identified from the primary miRNA mimics screening (Table 1), we compared the phenotypes with the miRNA inhibitor data. Following miRNA inhibitor experiments, hits that did not result in opposing phenotypes when compared to their mimic counterparts were excluded. The miRNA mimics which lead to cell death were also excluded. We also identified 29 miRNA mimics that when transfected into cells lead to decreased poliovirus replication (Fig. 5). From this data, miRNA-134 was selected for further investigation in this study.

### Polio Type II specific antibody-capture ELISA

A PV type-II specific ELISA was used to determine miRNA effects on Sabin-2 replication. Antibody-capture ELISA for detection of poliovirus was described previously^14,15^. Briefly, 2HB high-binding 96-well plates (NUNC; 1424561-PK) were coated with 50 ul of the PV type II specific monoclonal antibody (HYB294-06, mouse; Thermo Scientific Pierce) diluted in carbonate-bicarbonate buffer (pH 9.6). Plates were incubated for 16 h (overnight) at 4 °C in a moist chamber. Antibody-coated plates were washed with 0.05% phosphate buffered saline with Tween 20 (PBST). Following washing, plates were blocked with 100 μl blocking buffer (1x PBS containing 0.5% Gelatin and 0.25% Tween) for 60 min at 37 °C. Block was removed and plates were washed 4x with PBST. Fifty microliters of cell lysate was transferred from the thawed infected transfection plate to the antibody-coated plates. Inoculated plates were incubated for 60 min at 37 °C in a moist chamber. Subsequently, plates were washed 4x with PBST and 50 ul of HRP-substrate solution (SureBlue TMB) was added and incubated for 15 min at 37 °C. The reaction was halted by the addition of 50 ul H_2_SO_4_ stop solution. Absorbance was evaluated at a wavelength of 450 nm with a plate spectrophotometer (Tecan). Absorbance values were normalized to non-targeting miRNA mimic controls and converted to a z-score. Z-scores were used to determine whether hits are positive according to methods describe previously^20^. Hits with a z-score≤−1.5 s.d. were subjected to validation experiments in HEp-2C cells.

### Poliovirus (PV) and enterovirus 71 (EV71) plaque assays

PV and EV71 viral titers were determined post-transfection by either a HEp-2 or RD cell-based plaque assay, respectively^19,21^. Briefly, 1.2×10^6^ cells were seeded in each well of six well plates and incubated overnight under standard cell culture conditions. The next day culture media was removed and cells were washed with minimal essential medium (MEM; Hyclone, GE Healthcare, USA) supplemented with 2% fetal bovine serum (FBS). Next, samples were serially diluted 10-fold in MEM with 2% FBS and added to washed plates. Plates were incubated at 37 °C, 5% CO_2_ for 1 h. The inoculum was removed and plates were overlaid with 0.9% nutrient agarose gel in MEM supplemented with 2% FBS, followed by incubation in a 37 °C incubator with 5% CO_2_ for 3 days. Agarose overlay was removed and cells fixed with 10% formaldehyde in PBS. Plaques were visualized by staining with crystal violet. Virus titers were expressed as PFU ml^−1^.

### RNA isolation and qRT-PCR

Cells were homogenized and total RNA was extracted using RNAzol RT reagent (Molecular Research Center) according to the manufacturer’s instruction. The concentration of the purified RNA was determined by NanoDrop spectrophotometer (Thermo Scientific). To examine mature miR-134-5 expression, 300 ng total RNA was reverse-transcribed to cDNA using the miRNA 1st-Strand cDNA synthesis kit (Agilent Technologies) according to the manufacturer’s protocol. For the RAN gene, 300 ng total RNA was reverse-transcribed to cDNA using the AffinityScript QPCR cDNA synthesis kit (Agilent Technologies). For the quantification of individual genes, cDNAs were diluted in water and quantified using Brilliant III Ultra-Fast SYBR Green QPCR Master Mix (Agilent Technologies) in an Agilent Mx3005P instrument. Primers specific for miR-134-5p (forward, 5′-TGTGACTGGTTGACCA-3′; reverse, universal primer) RAN (forward, 5′-GAAGCTCATTGGAGACCCTAAC-3′; reverse, 5′-CAACCTCTAAGTCGTGCTCATAC-3′) were used. The gene expression levels were normalized to 18s rRNA gene as an internal control.

### Ingenuity pathway analysis and TargetScan analysis

QIAGEN’s Ingenuity pathway analysis (IPA; QIAGEN Redwood City; www.qiagen.com/ingenuity) was utilized to evaluate protein networks associated with PV replication in an effort to predict targets of miR-134 that may be involved in poliovirus infection and replication. TargetScan analysis (Whitehead Institute for Biomedical Research; http://www.targetscan.org/) was used to predict target transcripts via seed region complementarity. Pathway analysis and TargetScan analysis revealed many predicted targets, of interest was the nuclear transport system protein (RAN) because it has been previously published that another microRNA, miR-197, inhibited enterovirus 71 replication by targeting the RAN protein^16^.

### Data normalization and statistical analysis

The current screen was examined using Z’-factor (1>Z’>0.5: Excellent assay; 0.5>Z’>0: acceptable) to evaluate its quality. The majority of our screen fell into a Z’ factor (0.5<Z’<1.0) which reflects high-quality of the primary screen. Afterwards, the primary screen data was normalized to the entire plate and generated the mean (μ) of the data to zero and the standard deviation (s.d.) to 1. To achieve a high-quality and robust screen, we used siTOX (GE Healthcare Dharmacon TOX™ Transfection Control, Cat. # D0015000120) to monitor the transfection efficiency; we also used a siRNA targeting poliovirus (CDC, provided by Dr Steve Oberste) to monitor silencing effects on poliovirus replication (see transfection control section). A non-targeting siRNA (GE Healthcare Dharmacon: Cat. # D-001810-0X) used as a negative control. All experiments were performed in at least two independent replicates with each experiment consisting of duplicates for each miRNA targeting a specific mRNA transcript for the gene of interest. ELISA absorbance values were normalized by correcting the raw data. The mean of the replicates where calculated and standardized using Z-score analysis. The mean of the wells treated with experimental miRNAs for each miRNA were compared to the mean of negative control treated wells. The Z-score was calculated by finding the difference between the normalized score and the mean of the population and dividing this by the standard deviation of the population. Positive hits from the primary screen are scored by Z-score>1.5 s.d.

All results are presented as means±s.e. Statistical analysis was done using the one-way ANOVA with Turkey’s multiple comparison at 95% confidence level. *P*-values<0.05 were considered statistically significant.

## Data Records

### Data record 1

The miRNA mimic screen data presented is available at PubChem under *NCBI PubChem BioAssay* AID 1224906 (Data Citation 1). In this RNAi screen, miRNA mimics corresponding to 1,208 known miRNAs in the human genome were screened in HEp-2 cells for their regulatory effects against Sabin-2 poliovirus. One hundred eight miRNAs resulted in a substantial reduction of poliovirus replication relative to non-targeting control (z-scores≤−1.5). Forty-six miRNAs resulted in a substantial increase of poliovirus replication relative to non-targeting control (z-scores≥1.5) (Table 2). Included within this dataset are the hits which resulted in a standard z-score≤−0.5 and z-score≥0.5 compared to non-targeting control.

### Data record 2

The miRNA inhibitor data is available at PubChem under *NCBI PubChem BioAssay* AID 1224851 (Data Citation 2). The top miRNA hits from the mimic screening assays were re-screened in HEp-2 cells, to eliminate false-positive hits. In this case, miRNA inhibitors were tested to determine if they induced a phenotype which correlated with that of the miRNA mimic results. Included within this dataset are the z-scores of all miRNAs inhibitors tested.

## Technical Validation

### Transfection optimization

To establish a highly efficient miRNA transfection protocol in a high-throughput screening (HTS) fashion, we first investigated four individual DharmaFECT transfection reagents (DF1-4). We identified that DF4 is the most effective in gene silencing for both HEp-2. Further we performed the titration of the concentration of DF4 in both cell lines. In the case with 0.3% DF4 concentration, the GAPDH silencing was achieved >90%, and cell toxicity was not observed in either microscopy examination or MTT assay.

### Cytotoxicity assays

Cytotoxicity as a result of miRNA transfection was evaluated by CytoTox-Glo assay (Promega) according to manufacturer instructions. Briefly, HEp-2 cells were reverse transfected with miRNA mimics as described previously^22^. Forty-eight hours post-transfection cellular toxicity was evaluated. Values were normalized to siTOX control (100% cytotoxic). MiRNAs which elicited greater than 20% toxicity were excluded as is standard practice. In this study, we focused on the miRNA mimics screening, and observed some miR-induced cell death. Toxic miRNAs that lead to cell death including miR-1275, miR-129-3P, miR509, miR-1909, miR323-5P, miR-4265, miR-3661 were excluded from further studies. For the list of hits identified from miRNA mimics screening, we did not observe significant cell death for the corresponding miRNA inhibitors.

### Transfection controls

A high-quality RNAi screen integrates both excellent experimental design and computational strategies for quality control. A clear distinction between a positive control and a negative reference is paramount. In the current miRNA screen in HEp-2 cell line, a positive control siRNA specifically targeting poliovirus (Poliovirus-specific (VP1 & 3D) and a negative control (non-targeting siRNA) were clearly distinguished from each other in all 96-well plates transfected with siRNAs. siTOX (GE Healthcare Dharmacon TOX Transfection Control, Cat. # D0015000120) was used to serve as another indicator for transfection efficiency and a mock control used as background normalization.

**Figure 6:**
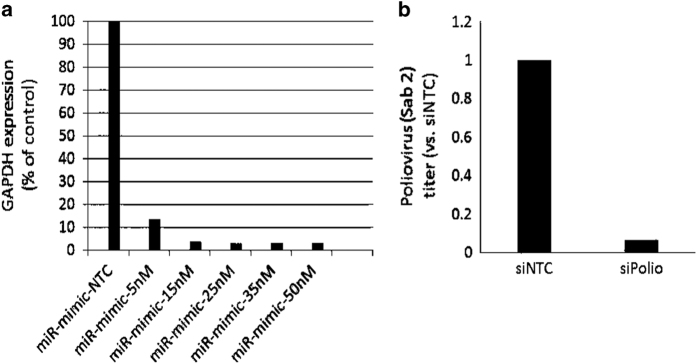
(a) optimization of transfection using miRIDIAN microRNA mimic housekeeping positive control #2 (GAPDH) (GE Dharmacon; Cat # CP-001000-02). (**b**) Optimization of positive control (siPol) for virus replication (CDC, provided by Dr Steve Oberste).

As part of developing a screening assay with low variation and a strong signal to background ratio we conducted pilot studies to optimize screening conditions. These conditions included optimizing titration of miRNA and siRNAs used to determine the most effective concentrations. Assay responses to negative and positive miRNA and siRNA controls were also evaluated and optimized to determine baseline values and standard deviation value for the calculation of Z-scores. Z-scores were subsequently used to determine hits for further screening. Transfection efficiency was optimized via transfection with miRIDIAN microRNA Mimic housekeeping positive Control #2 (GAPDH) (Cat. # CP-001000-02) as well as the inclusion of siTOX transfection control as previously mentioned. MRNA transcription levels were evaluated by qPCR following miRNA transfection and compared to GAPDH control to confirm miRNA mediated mRNA knockdown. Positive controls for poliovirus screening were carefully selected and optimized (Fig. 6)^23^. The culmination of this data suggests high transfection efficiency, low variability and substantial positive control values concluding that our screening procedure is robust.

## Additional Information

**How to cite this article**: Orr-Burks, N. L. *et al.* MicroRNA screening identifies miR-134 as a regulator of poliovirus and enterovirus 71 infection. *Sci. Data* 4:170023 doi: 10.1038/sdata.2017.23 (2017).

**Publisher**’**s note**: Springer Nature remains neutral with regard to jurisdictional claims in published maps and institutional affiliations.

## Supplementary Material



## Figures and Tables

**Table 1:** Top anti-PV miRNA hits.

**miRNA**	**PreCursor Accession**	**Normalize Z-score**	**miRNA**	**PreCursor Accession**	**Normalize Z-score**	**miRNA**	**PreCursor Accession**	**Normalize Z-score**
hsa-let-7i*	MI0000434	−1.6437	hsa-miR-29b	MI0000105	−2.0564	hsa-miR-432	MI0003133	−1.6267
hsa-miR-1181	MI0006274	−1.5697	hsa-miR-29c	MI0000735	−1.8369	hsa-mir-4326	MIMAT0016888	−1.5294
hsa-miR-1182	MI0006275	−1.7367	hsa-miR-301b	MI0005568	−1.7121	hsa-miR-450b-3p	MI0005531	−1.6297
hsa-miR-1207-5p	MI0006340	−1.7136	hsa-miR-30c	MI0000736	−1.5034	hsa-miR-483-5p	MI0002467	−1.8311
hsa-miR-1227	MI0006316	−1.821	hsa-mir-3118-1	MIMAT0014980	−1.8229	hsa-miR-491-5p	MI0003126	−1.8349
hsa-mir-1244-2	MI0015974	−1.6289	hsa-mir-3123	MIMAT0014985	−1.5285	hsa-miR-497	MI0003138	−1.5982
hsa-miR-1250	MI0006385	−2.173	hsa-mir-3127	MI0014144	−1.9938	hsa-miR-509-3p	MI0005717	−2.185
hsa-miR-1255b	MI0006435	−1.7509	hsa-mir-3132	MIMAT0014997	−2.0313	hsa-miR-516b	MI0003172	−1.7892
hsa-miR-1257	MI0006391	−2.0261	hsa-mir-3140	MI0014163	−1.8811	hsa-miR-517b	MI0003165	−1.5992
hsa-miR-125b	MI0000446	−1.7329	hsa-mir-3175	MIMAT0015052	−1.5215	hsa-miR-518a-5p	MI0003170	−1.5166
hsa-miR-1274a	MI0006410	−1.5221	hsa-mir-3186	MI0014229	−1.7061	hsa-miR-518e	MI0003169	−1.605
hsa-miR-1275	MI0006415	−1.8306	hsa-mir-3190	MIMAT0015073	−1.7578	hsa-miR-522	MI0003177	−1.8432
hsa-miR-129-3p	MI0000473	−1.7159	hsa-mir-3191	MI0014236	−1.6572	hsa-miR-523	MI0003153	−1.617
hsa-miR-129*	MI0000252	−1.8363	hsa-mir-3192	MIMAT0015076	−2.0036	hsa-miR-544	MI0003515	−1.6514
hsa-miR-1293	MI0006355	−1.7672	hsa-mir-3194	MI0014239	−1.721	hsa-miR-545*	MI0003516	−1.8565
hsa-miR-1298	MI0003938	−1.5262	hsa-miR-320d	MI0008190	−1.5835	hsa-mir-548aa-1	MIMAT0018447	−1.9087
hsa-miR-130a	MI0000448	−1.6326	hsa-miR-323-5p	MI0000807	−1.8961	hsa-miR-548n	MI0006399	−1.5612
hsa-miR-134	MI0000474	−2.3096	hsa-miR-324-5p	MI0000813	−1.5969	hsa-miR-555	MI0003561	−2.0986
hsa-miR-138	MI0000476	−2.4403	hsa-miR-330-5p	MI0000803	−1.8413	hsa-miR-588	MI0003597	−1.677
hsa-miR-149*	MI0000478	−1.6051	hsa-miR-338-5p	MI0000814	−1.8383	hsa-miR-597	MI0003609	−1.5576
hsa-miR-15b*	MI0000438	−1.6419	hsa-miR-342-5p	MI0000805	−1.898	hsa-miR-608	MI0003621	−1.744
hsa-miR-16	MI0000070	−1.9572	hsa-miR-362-5p	MI0000762	−1.5916	hsa-miR-609	MI0003622	−1.7592
hsa-miR-1908	MI0008329	−1.5895	hsa-miR-363	MI0000764	−1.6293	hsa-miR-613	MI0003626	−1.7465
hsa-miR-1909	MI0008330	−1.8725	hsa-mir-3661	MIMAT0018082	−1.7807	hsa-miR-622	MI0003636	−2.0702
hsa-miR-1914*	MI0008335	−1.6261	hsa-mir-3691	MI0016092	−1.5575	hsa-miR-624	MI0003638	−1.6856
hsa-miR-195	MI0000489	−1.7648	hsa-miR-381	MI0000789	−1.6366	hsa-miR-628-5p	MI0003642	−1.8112
hsa-miR-197	MI0000239	−1.8252	hsa-mir-3915	MIMAT0018189	−1.5228	hsa-miR-630	MI0003644	−1.8071
hsa-miR-218-2*	MI0000295	−1.5057	hsa-miR-411*	MI0003675	−1.7965	hsa-miR-663	MI0003672	−1.6806
hsa-miR-220c	MI0005536	−1.6914	hsa-miR-421	MI0003685	−1.7236	hsa-mir-676	MI0016436	−1.5983
hsa-miR-224*	MI0000301	−1.8876	hsa-miR-424	MI0001446	−1.7503	hsa-miR-7-2*	MI0000264	−2.0278
hsa-mir-2278	MIMAT0011778	−1.5577	hsa-mir-4252	MIMAT0016886	−1.5233	hsa-miR-768-3p	MI0005117	−1.6025
hsa-mir-2355	MI0015873	−1.5942	hsa-mir-4255	MIMAT0016885	−1.5652	hsa-miR-769-3p	MI0003834	−1.977
hsa-miR-26a	MI0000083	−1.6853	hsa-mir-4265	MIMAT0016891	−1.6974	hsa-miR-801	MI0005202	−1.561
hsa-miR-26b	MI0000084	−1.7231	hsa-mir-4270	MIMAT0016900	−1.5457	hsa-miR-922	MI0005714	−1.5015
hsa-miR-27a*	MI0000085	−1.9841	hsa-mir-4292	MIMAT0016919	−1.5023	hsa-miR-924	MI0005716	−1.5341
hsa-miR-29a	MI0000087	−1.8126	hsa-miR-431*	MIMAT0004757	−1.8885	hsa-miR-92a-1*	MI0000093	−1.5144
Listed are the top 108 miRNA (z-score≤−1.5) which reduced Sabin-2 replication following transfection.								

**Table 2:** Top pro-PV miRNA hits.

**miRNA**	**PreCursor Accession**	**Normalized Z-score**	**miRNA**	**PreCursor Accession**	**Normalized Z-score**
hsa-miR-106a	MI0000113	2.5029	hsa-mir-3622b	MI0016014	2.8955
hsa-miR-1183	MI0006276	2.2776	hsa-mir-3650	MIMAT0018070	3.0868
hsa-miR-1256	MI0006390	2.2043	hsa-miR-371-3p	MIMAT0000723	3.0328
hsa-mir-1270-2	MI0006407	1.8131	hsa-mir-3913-1	MI0016417	1.8771
hsa-miR-1290	MI0006352	2.1255	hsa-miR-411	MI0003675	1.6903
hsa-miR-148b	MI0000811	2.1563	hsa-miR-429	MIMAT0001536	2.3367
hsa-miR-182	MI0000272	1.813	hsa-mir-4300	MIMAT0016853	2.2905
hsa-miR-1825	MI0008193	1.6288	hsa-mir-4311	MIMAT0016863	1.8049
hsa-miR-185*	MI0000482	2.5167	hsa-miR-488	MI0003123	1.7722
hsa-miR-190b	MI0005545	1.5325	hsa-miR-512-3p	MI0003140	2.4828
hsa-miR-1977	MI0009987	2.8377	hsa-miR-513a-5p	MI0003191	1.6767
hsa-miR-1978	MI0009988	2.033	hsa-miR-519c-3p	MI0003148	1.5004
hsa-mir-2115*	MIMAT0011159	2.2428	hsa-miR-519d	MI0003162	1.789
hsa-miR-216a	MI0000292	1.5712	hsa-miR-520c-3p	MI0003158	2.1928
hsa-miR-22	MI0000078	1.6283	hsa-miR-520d-3p	MI0003164	2.3981
hsa-miR-221*	MI0000298	2.2304	hsa-miR-520e	MI0003143	1.7425
hsa-mir-3155	MIMAT0015029	1.7369	hsa-miR-526b*	MI0003150	2.2129
hsa-mir-3160-1	MI0014189	2.0883	hsa-miR-559	MI0003565	1.8168
hsa-mir-3162	MI0014192	2.7709	hsa-miR-571	MI0003578	1.9588
hsa-mir-3187	MI0014231	2.0815	hsa-miR-576-3p	MI0003583	2.4894
hsa-mir-3605	MI0015995	1.7167	hsa-miR-589	MI0003599	2.0177
hsa-miR-361-5p	MI0000760	2.295	hsa-miR-598	MI0003610	1.5474
hsa-mir-3620	MI0016011	2.5204	hsa-miR-9	MI0000466	2.1298
Listed are the top 46 miRNA (z-score≥1.5) which increased Sabin-2 replication following transfection.					
